# Community Cultural Norms, Stigma and Disclosure to Sexual Partners among Women Living with HIV in Thailand, Brazil and Zambia (HPTN 063)

**DOI:** 10.1371/journal.pone.0153600

**Published:** 2016-05-06

**Authors:** Bisola O. Ojikutu, Subash Pathak, Kriengkrai Srithanaviboonchai, Mohammed Limbada, Ruth Friedman, Shuying Li, Matthew J. Mimiaga, Kenneth H. Mayer, Steven A. Safren, HIV Prevention Trials Network 063 Team

**Affiliations:** 1 Division of Global Health Equity, Brigham and Women’s Hospital, Boston, Massachusetts, United States of America; 2 Harvard Medical School, Boston, Massachusetts, United States of America; 3 Statistical Center for HIV/AIDS Research and Prevention, University of Washington, Seattle, Washington, United States of America; 4 Faculty of Medicine, Chiang Mai University, Chiang Mai, Thailand; 5 Research Institute for Health Sciences, Chiang Mai University, Chiang Mai, Thailand; 6 University of Zambia, Lusaka, Zambia; 7 University of California Los Angeles, Los Angeles, California, United States of America; 8 Brown University School of Public Health, Providence, Rhode Island, United States of America; 9 The Fenway Institute, Boston, Massachusetts, United States of America; 10 University of Miami, Miami, Florida, United States of America; University of Toronto, CANADA

## Abstract

**Background:**

Serostatus disclosure may facilitate decreased HIV transmission between serodiscordant partners by raising risk awareness and heightening the need for prevention. For women living with HIV (WLWH), the decision to disclose may be influenced by culturally determined, community-level stigma and norms. Understanding the impact of community HIV stigma and gender norms on disclosure among WLWH in different countries may inform intervention development.

**Methods:**

HPTN063 was a longitudinal, observational study of sexually active HIV-infected individuals, including heterosexual women, in care in Zambia, Thailand and Brazil. At baseline, a questionnaire measuring community HIV stigma and gender norms, anticipated stigma, demographic, partner/relationship characteristics, and intimate partner violence was administered. Longitudinal HIV disclosure to sexual partners was determined via audio-computer assisted self-interview (ACASI) at the baseline and quarterly during the one year following up. Logistic regression was conducted to identify the predictors of disclosure.

**Results:**

Almost half (45%) of women living with HIV acknowledged *perceived community HIV stigma* (the belief that in their community HIV infection among women is associated with sex work and multiple sexual partners). Many women (42.9%) also acknowledged *perceived community gender norms* (the belief that traditional gender norms such as submissiveness to husbands/male sexual partners is necessary and that social status is lost if one does not procreate). HIV disclosure to current sex partners was reported by 67% of women. In multivariate analysis, among all women, those who were older [OR 0.16, 95%CI(0.06,0.48)], reported symptoms of severe depression [OR 0.53, 95%CI(0.31, 0.90)], endorsed *anticipated stigma* [OR 0.30, 95%CI(0.18, 0.50)], and were unmarried [OR 0.43, 95%CI(0.26,0.71)] were less likely to disclose to current partners. In an analysis stratified by marital status and cohabitation, unmarried [OR 0.41, 95%CI(0.20,0.82)] and non-cohabiting women [OR 0.31, 95%CI(0.13,0.73)] who *perceived community HIV stigma* were less likely to disclose to their sex partners.

**Conclusions:**

*Perceived community level HIV stigma*, along with individual level factors such as *anticipated stigma*, depressive symptoms, and older age, predict non-disclosure of HIV status to sexual partners among WLWH in diverse geographic settings. Interventions to promote disclosure among women in serodiscordant relationships should incorporate community-level interventions to reduce stigma and promote gender equality.

## Introduction

Among women living with HIV, approximately 50% are in serodiscordant sexual relationships.[1,2] Within such relationships, HIV status disclosure has proven effective in decreasing transmission to the HIV-negative partner by raising risk awareness and heightening the need for HIV prevention, including routine counseling and testing, pre-exposure prophylaxis, and condom use. [3,4] Though voluntary disclosure is a recommended strategy, people living with HIV are often unaware of their partner’s status and have not shared their own. [5–9] Interventions designed to increase disclosure among heterosexual couples, such as couples based counseling and testing, have been developed and implemented with moderate success. [10–12]

Recent research on disclosure in heterosexual relationships has highlighted the unique barriers faced by women living with HIV. [7, 13–16] Early in the epidemic, it was assumed that the male partner was the “index” case (the person from whom HIV is acquired), and therefore bore the burden of disclosing to his female partners. [17] Recognizing that women are often the HIV-infected partner, studies have explored gender-based differences in disclosure patterns, particularly in the context of pregnancy. [18,19] While data remain inconclusive, several studies have also noted that women living with HIV are less likely to disclose than men. [20,21] Irrespective of gender, the decision to disclose is complex and likely contingent upon an estimation of future consequences. [22] An individual living with HIV must weigh the risk of potential harm to themselves following disclosure versus the potential benefit to their sexual partners. For a woman, the consequences of disclosure may be dire. Gender inequality and relationship power dynamics contribute to an increased likelihood among women to experience negative consequences of disclosure. [23] Following disclosure, many women have reported economic loss, abandonment and intimate partner violence. [24,25]

Community-held, socially shared norms and beliefs may influence a woman’s decision making process regarding status disclosure. Community beliefs may devalue individuals living with HIV and potentiate *felt normative stigma* or the subjective awareness of HIV stigma in one’s local community. [26] *Felt normative stigma* (henceforth called “perceived community HIV stigma”) may influence a woman’s decision making process regarding disclosure. [27,28] Community beliefs and societal norms may also perpetuate gender inequality, thus shaping communication within intimate relationships and dictating whether and when a woman feels free to disclose. [29] Cultural emphasis on motherhood, personal desire for child-bearing, and normative marital behavioral standards may impede a woman’s ability to disclose and promote the use of condoms. [30–32] Congruence between a woman’s own beliefs and these community-level cultural beliefs may promote *anticipated stigma* (the fear of being devalued or discriminated against by others if one’s HIV positive becomes known) and lead to non-disclosure. [33]

Because disclosure is a complex process, behavioral health interventions should reflect an understanding of the myriad factors that impact its likelihood, including community-level norms and beliefs as well as individual-level relationship and life circumstances, psychosocial issues, mental health and clinical status. Few studies have explored the role of multi-level factors on disclosure patterns among women from a cross-cultural perspective. Most have focused solely on women living with HIV in sub Saharan Africa and have explored individual-level predictors. The community-level norms and beliefs and individual-level socio-demographic factors that may affect a women’s decision to disclose are likely to be contextual and may vary widely within and between countries. The current study explores disclosure patterns of women living with HIV in three geographic settings representative of concentrated and generalized epidemics: Brazil, Thailand and Zambia. Determining factors that predict disclosure to sexual partners among women living in diverse settings may lead to more effective interventions to decrease sexual transmission risk and improved quality of life among women living with HIV.

## Methods

### Study Design

HPTN 063 was a multi-site, observational cohort study of sexually active people living with HIV in care in Africa (Zambia), Asia (Thailand), and South America (Brazil) which was designed to collect data to assist in the development of interventions to decrease sexual transmission risk behaviors in people living with HIV and to determine whether similar interventions could be used across various sexual risk groups and cultural settings. Adult women ages 18 and over were enrolled in HPTN063 if they were HIV-infected, receiving care (attended at least two visits to an HIV clinic in the nine months prior to the study), and reported recent (within 3 months) HIV transmission risk behavior including unprotected receptive anal or vaginal intercourse (i.e. without a condom) with a person who is HIV uninfected or of unknown HIV status. A total of 299 heterosexual women were enrolled. Additional details regarding study design, recruitment and enrollment have been described. [34]

### Perceived Community HIV Stigma and Community Gender Norms

At baseline, a survey was administered by trained research assistants in the participant’s preferred language. Perceived community beliefs were assessed via 23 items included in the baseline questionnaire. Participants were asked to indicate agreement with statements prefaced by the phrase “In my community…” to capture their perception of community beliefs. Responses were measured on a 4-point Likert scale (disagree strongly, disagree, agree, strongly agree). Principal component analysis (PCA) was used to assess the underlying structure and reduce the dimensions of questionnaire items. The number of principal components was determined by eigenvalues greater than one, the proportion of variation explained, and the interpretations of the eigenvalues. The varimax orthogonal rotation option was used to obtain distinct principal components. Two components were obtained from PCA analysis: *perceived community HIV stigma* and *perceived community gender norms*. The first component, *perceived community HIV stigma*, accounted for 35% of the variance, and the second component, *perceived community gender norms*, accounted for 21% of the variance. *Perceived community HIV stigma* had a higher loading in three questionnaire items which focused on sexual mores: (1) People believe that people with HIV have contracted the disease because they have engaged in immoral behavior; (2) People believe that women with HIV have engaged in prostitution; and (3) People believe that women with HIV have engaged in sex with many partners. *Perceived community gender norms* had a higher loading in three questionnaire items which focused on marriage and procreation: (1) Women are obligated to be submissive to their husbands, and therefore cannot ask their husbands to use condoms; (2) There is an obligation to have children and therefore engage in unprotected (condomless) sex; and (3) I will lose my status in my community if I am not a mother or a father. The loadings for the two components are provided S1 Table. For each of the components, standardized scores centered at zero were calculated and dichotomized as positive (agree) vs. negative (disagree).

### Anticipated Stigma

Anticipated stigma or the fear of consequences of disclosure was assessed via 5 items included within the baseline survey: (1) I fear discrimination if I disclose my HIV positive status to others; (2) I fear being kicked out of my community if I disclose my HIV status to others; (3) I am afraid of violence if I disclose my HIV positive status to others; (4) I am afraid of losing my job if I disclose my HIV status to my boss or others; and (5) I fear being kicked out of my house if I disclose my HIV positive status to others. Similar measures have been used to assess anticipated stigma in other studies. [35,36] Responses were on a 4-point Likert scale (strongly disagree, disagree, agree, strongly agree). One component measuring anticipated stigma using these 5 items was obtained by PCA using the criteria of eigenvalue greater than one, the proportion of variation explained, and the interpretations of the eigenvalues. Slightly higher than half of the variance was accounted for by this component. The loadings of each item in this component are provided S2 Table. The component score was standardized and dichotomized as positive or negative. The Cronbach alpha for these items is .74.

### Other Independent Variables

The baseline survey collected demographic characteristics including age, marital status, highest level of education, employment and gender identity. Employment was assessed by asking participants to indicate their current work situation by selecting from a list of twenty paid and unpaid occupations including housework and sex work. For gender identity, participants were asked “Do you consider yourself to be transgender?” Different community and social norms may drive disclosure decisions among transgender individuals. Therefore, transgender individuals were not included in this analysis. Depressive symptoms were assessed by the Center for Epidemiologic Studies Depression Scale (CES-D), a 20 item measure of depressive symptoms validated in international settings.[37] The range of CES-D scores is 0–60 with a score of <7 indicating no depressive symptoms and 7 to 16 indicating mild to moderate depressive symptoms. A score greater than 16 is indicative of significant or severe depressive symptoms. [38] Number of current sexual partners and primary partner’s HIV status (positive, negative or unknown) were obtained. Women were also asked whether or not they were currently living with their primary sexual partner (cohabitation status) and whether or not they desired additional biological children. History of alcohol use was measured using the Alcohol Use Disorders Identification Test (AUDIT-10), a 10-item screen for alcohol use disorders which has been validated in international settings. [39] Total scores of 8 or more are recommended as indicators of hazardous and harmful alcohol use, as well as possible alcohol dependence. [40] A list of locally used substances at each site was used to determine history of illicit drug use. History of recent and past intimate partner violence (IPV) was also assessed. Clinical factors, antiretroviral therapy prescription and adherence, were obtained by participant self-report. A specimen was collected for CD4 count measurement.

### Outcome

Disclosure to sexual partners, sexual risk behavior and clinical status were assessed at baseline and at 3, 6, 9 and 12 months via audio computer assisted survey (ACASI) during one year of follow-up. For disclosure participants were asked “Thinking about the people you have had vaginal or anal sex with in the last 3 months how often have you told them that you are HIV positive?” Seven responses were possible (1) I have not had vaginal or anal sex in the last 3 months; (2) They already knew that I was HIV positive; (3) I did not tell any of them; (4) I told less than half; (5) I told about half; (6) I told more than half; (7) I told all of them. Responses were aggregated to create a binary variable (disclosed to any vs. disclosed to none).

### Statistical Analysis

To test differences in the demographic and baseline characteristics among the sites, we used Chi-square test for categorical variables and ANOVA for continuous variables. To determine the predictor(s) for the disclosure outcome collected from ACASI over time, we used the Generalized Estimating Equations (GEE) method with an assumption of binomial distribution and logit link for the dichotomized outcome collected over time. Inverse probability weighting was used to account for disproportional missing observations in outcomes across sites over time and robust standard error estimate was used in the inference to account and control the correlations of the outcomes within individuals. All clinical, socio-demographic and psychosocial variables were evaluated in the univariate models and the variables with p<0.1 in the univariate analysis were evaluated jointly in the multivariate model. Sites and visit times were adjusted for in both the univariate and the multivariate models. The associations of stigma and community norms with the disclosure outcomes were also evaluated in models stratified by marital or cohabitation status. All statistical analyses were performed using SAS 9.4.

### Ethical Approval

This study was approved by institutional review boards within each country. The Research Institute for Health Sciences at Chiang Mai University and the Johns Hopkins Bloomberg School of Public Health approved the study in Thailand. In Zambia, the study was approved by the Zambian Ministry of Health, the University of Zambia, and the University of Alabama at Birmingham. In Brazil, the study was approved by the Evandro Chagas Clinical Research Institute, and the National Committee for Ethics in Research. Written informed consent was obtained from each participant. Each IRB approved this consent procedure.

## Results

### Demographics

A total of 299 women were included in the study at baseline, 100 in Thailand, 100 in Zambia and 99 in Brazil (Table 1). The median age of participants was 38 (IQR 33, 43). The median number of current sex partners at baseline reported by Thai and Zambian women was 2 (IQR 1, 3) versus 1 (IQR 1,2) among Brazilian women (p = 0.0001). Fewer Brazilian women (83%) compared to Thai (94%) or Zambian (98%) women reported their partner’s status as negative or unknown (p = 0.0007). Brazilian women (75.8%) were more likely to be unmarried than women from Thailand (33%) or Zambia (31%) (p<0.0001). Most women (72%) were cohabiting with their primary sexual partner. Only 4 women (all living in Zambia) were pregnant at the time of the baseline assessment. More women living in Zambia (32%) than those living in Thailand (2%) or Brazil (17%) reported a desire for additional biological children (p<0.0001).

**Table 1:** Baseline socio-demographic characteristics of HPTN 063 female study participants by site.

	Total N(%)	Thailand N(%)	Brazil N(%)	Zambia N(%)	*p* value
**Total**	299(100)	100(33)	99(33)	100(33)	
Age (in years)					0.27
18–24	11(4.0)	3(3.0)	5(5.0)	3(3.0)	
25–44	232(78.0)	72(72.0)	81(82.0)	79(79.0)	
≥45	56(19.0)	25(25.0)	13(13.0)	18(18.0)	
**Relationship Characteristics**					
Median Number of Regular Sexual Partners (IQR)*	2(1,3)	2(1,3)	1(1,2)	2(1,3)	0.0001
Primary Sexual Partner HIV Status					0.0007
Negative or Unknown	254(92.0)	89(94.0)	68(83.0)	97(98.0)	
Positive	22(8.0)	6(6.0)	14(17.0)	2(2.0)	
Unmarried	139(46.5)	33(33.0)	75(75.8)	31(31.0)	<0.0001
Cohabiting with Primary Sexual Partner	198(72.0)	73(77.0)	54(66.0)	71(72.0)	0.27
Currently Pregnant	4(1.4)	0(0)	0(0)	4(4.0)	0.023
Desire for Additional Biological Children	51(17.0)	2(2.0)	17(17.0)	32(32.0)	<0.0001
**Social History and Mental Health**					
Highest Level of Education					0.013
Primary school	156(52.0)	58(58.0)	54(55.0)	44(44.0)	
Unemployed	134(45.0)	16(16.0)	40(40.0)	78(78.0)	<0.0001
Past History of Intimate Partner Violence	126(42.0)	38(38.0)	55(56.0)	33(33.0)	0.0019
Recent History of Intimate Partner Violence	21(7.0)	7(7.0)	6(6.0)	8(8.0)	0.90
Alcohol Abuse /Illicit Drug Use	37(12.0)	12(12.0)	22(22.0)	3(3.0)	0.002
Depression					
Severe Symptoms	104(35.0)	35(35.0)	46(47.0)	23(23.0)	0.0013
Mild to Moderate Symptoms	92(31.0)	29(29.0)	32(33.0)	31(31.0)	
**Clinical Variables**					
On Antiretroviral Therapy	262(88.0)	96(96.0)	78(79.0)	88(89.0)	0.0001
Adherence (Good, Very Good, Excellent)	223(88.0)	80(83.0)	57(81.0)	86(98.0)	0.009
CD4+ cell count (Mean cells/mm^3^)	568	457	730	525	<0.0001

*Interquartile range

Fewer women in Zambia (44%) completed primary school than women living in Thailand (58%) or Brazil (55%) (p = 0.013). Zambian women (78%) were more likely to be unemployed than Thai (16%) or Brazilian participants (40%) (p<0.0001). None of the women reported that they were sex workers. More Brazilian (56%) than Thai (38%) or Zambian (33%) women reported a past history of IPV (p = 0.0019). Only 7% of all women reported a recent history of IPV. Drug and alcohol abuse was reported more frequently among Brazilian (22%) than Thai (12%) or Zambian (3%) women (p = 0.002). Severe depressive symptoms were also reported more frequently by Brazilian than Thai women (47% vs 35%, p = 0.0013).

Fewer Brazilian women (79%) were taking antiretroviral therapy at baseline than Thai (96%) or Zambian (89%) women (p = 0.0001). More Zambian women (98%) reported good, very good or excellent adherence to treatment than Thai (83%) or Brazilian (81%) women (p = 0.009). The mean CD4 count among all women was 568 cells/mm^3^.

### HIV Stigma and Community Gender Norms

Nearly one-half of all women (45.3%) reported *perceived community HIV stigma* (sexual mores) at baseline (Table 2). No significant differences in the frequency of this type of stigma were noted across sites. At baseline, 42.9% of women also acknowledged *perceived community gender norms* (marriage and procreation). More Zambian women (66%) endorsed these norms than Thai (38%) or Brazilian (24%) women (p<0.0001). *Anticipated stigma* or the fear of the consequences of status disclosure was reported by 40.9% of all women. More Brazilian women (62.2%) reported *anticipated stigma* than women in Thailand (38%) or Zambia (23%) (p<0.0001).

**Table 2:** Perceived community stigma, perceived community norms and anticipated stigma among females participants of HPTN063 by site.

	Total N (%)	Thailand N (%)	Brazil N (%)	Zambia N (%)	*p* value
**Community-Level**					
Perceived Community Stigma	134(45.3)	42(42.0)	49(51.0)	43(43.0)	0.38
Perceived Community Norms	127(42.9)	38(38.0)	23(24.0)	66(66.0)	<0.0001
**Individual-Level**					
Anticipated Stigma	122(40.9)	38(38.0)	61(62.2)	23(23.0)	<0.0001

### Disclosure to Sexual Partners

Among all women, 67% reported disclosing to their sexual partner at baseline. No significant difference was noted in disclosure to sexual partners over time (baseline, 3, 6, 9 and 12 months) among the total group or within each site. No significant differences in disclosure were noted across sites.

In univariate analysis, women who were older (24–44 vs 18–24) [OR 0.30, 95%CI(0.12, 0.78)] and (≥45 vs 18–24) [OR 0.21, 95%CI (0.08, 0.60)], had symptoms of severe depression [OR 0.51, 95%CI (0.30,0.87)] and who reported *anticipated stigma* [OR 0.27, 95%CI(0.17,0.44)] were less likely to disclose (Table 3). Women who were unmarried [OR 0.37, 95%CI(0.23,0.59)] and those who were not cohabiting with their partner [OR 0.28, 95%CI(0.18, 0.46)] were also less likely to disclose to their sexual partners.

**Table 3:** Associations of female HPTN063 participant characteristics with disclosure to sexual partners.

	Univariate*		Multivariate†	
	OR(95%CI)	*p* value	OR(95%CI)	*p* value
Age (in years)				
25–44 vs 18–24	0.30(0.12, 0.78)	0.0128	0.20(0.07,0.56)	0.002
≥45 vs 18–24	0.21(0.08,0.60)	0.0034	0.16(0.06,0.48)	0.001
Primary Sexual Partner HIV Status				
Negative or Unknown vs Positive	0.73(0.37,1.44)	0.37	-	-
Unmarried vs. Married	0.37(0.23,0.59)	< .0001	0.43(0.26,0.71)	0.0007
Cohabitation with Primary Sex Partner (No vs Yes)ⱡ	0.28(0.18, 0.46)	< .0001	-	-
Desire for Additional Biological Children (Yes vs No)	1.04(0.59,1.85)	0.89	-	-
Highest Level of Education				
Primary school vs no schooling	0.21(0.02,1.89)	0.16	-	-
More than primary school vs no schooling	0.24(0.03,2.28)	0.22	-	-
Unemployed	1.25(0.75,2.07)	0.39	-	-
Past History of Sexual or Physical Abuse (Yes vs No)	1.24(0.81,1.92)	0.32	-	-
Recent History of Sexual or Physical Abuse (Yes vs No)	1.11(0.50,2.48)	0.79	-	-
Alcohol Abuse /Illicit Drug Use (Yes vs No)	1.00(0.52,1.95)	0.99	-	-
Depression				
Severe Symptoms vs None	0.51(0.30,0.87)	0.01	0.53(0.31,0.90)	0.02
Mild to Moderate vs None	0.80(0.46,1.38)	0.42	0.83(0.47,1.46)	0.51
On Antiretroviral Therapy (Yes vs No)	1.78(0.60,2.30)	0.64	-	-
Adherence (Good, Very good, Excellent)				
Good, Very good, excellent vs fair	0.69(0.32,1.53)	0.36	-	-
Good, Very good, Excellent vs Poor or Very poor	1.91(0.36,10.08)	0.44	-	-
CD4+ Cell Count	1.0(0.99,1.00)	0.74	-	-
Anticipated Stigma (Yes vs No)	0.27(0.17,0.44)	< .0001	0.30(0.18,0.50)	< .0001
Perceived Community Stigma (Yes vs No)	0.82(0.54,1.25)	0.36	0.85(0.54,1.31)	0.46
Perceived Community Norms (Yes vs No)	1.11(0.71,1.75)	0.64	1.47(0.90,2.42)	0.13

*Sites and visit times were adjusted for in both the univariate and the multivariate models.

^†^ Age, marital status, depression, anticipated stigma, perceived community stigma, perceived community norms, sites, and visit times were included in the multivariate model.

^ⱡ^ Cohabitation status was noted to be collinear with marital status and was removed from the multivariate model.

In the multivariate model which was adjusted for age, marital status, depression, *anticipated stigma*, *perceived community gender norms*, *perceived community HIV stigma*, site and visit number, women who were older (25–44 vs 18–24) [OR 0.20, 95%CI (0.07, 0.56)] and (≥45 vs 18–24) [OR 0.16, 95%CI(0.06,0.48)], who reported *anticipated stigma* [OR 0.30, 95%CI(0.18,0.50)], who noted symptoms of severe depression [OR 0.53, 95%CI(0.31,0.90)] and who were unmarried [OR 0.43, 95%CI(0.26,0.71)] remained less likely to disclose. Cohabitation was found to be collinear with marital status and was not included in the multivariate model.

### Cohabitation and Marital Status Modified the Associations

The associations of *perceived community HIV stigma*, *perceived community gender norms*, and *anticipated stigma* with disclosure were determined after stratifying by marital and cohabitation status (Table 4). In univariate analysis, among women who were not cohabiting with their sexual partner, those who acknowledged *perceived community HIV stigma* [(OR 0.28, 95%CI(0.12,0.67)] and *anticipated stigma*[OR 0.33, 95%CI (0.14,0.74)] were significantly less likely to disclose to their sexual partner(s). In multivariate analysis, both forms of stigma remained significant [*perceived community HIV stigma* [OR 0.36, 95%CI (0.13,0.73)] and *anticipated stigma* [OR 0.31, 95%CI (0.13,0.52)] among non-cohabiting women. Among unmarried women, those reporting *perceived community HIV stigma* [OR 0.53, 95%CI (0.29,0.97)] and *anticipated stigma* [OR 0.31, 95%CI (0.16,0.59)] were significantly less likely to disclose to their sexual partner(s) in univariate analysis. Both forms of stigma remained significant in multivariate analysis [*perceived community HIV stigma* [OR 0.41, 95%CI(0.20,0.82)] and *anticipated stigma* [OR 0.25,95%CI(0.11,0.57)]. Among both married and cohabiting women, participants reporting *anticipated stigma* were less likely to disclose in multivariate analysis (married: [OR 0.22, 95%CI(0.10,0.50)] and cohabiting: [OR 0.26, 95%CI (0.13,0.52)]. *Perceived community HIV stigma* and *perceived community gender norms* were not found to be significant predictors of disclosure among married or cohabiting women.

**Table 4:** Associations of anticipated stigma, perceived community stigma, and perceived community norms with disclosure to sexual partners.

	Univariate		Multivariate	
	OR (95%CI)	*p* value	OR(95%CI)	*p* value
**Non-Cohabiting Women**				
Perceived Community Stigma (Y vs N)	0.28(0.12,0.67)	0.004	0.31(0.13,0.73)	0.008
Perceived Community Norms (Y vs N)	1.36(0.61,3.02)	0.46	—	—
Anticipated Stigma (Y vs N)	0.33(0.14,0.74)	0.008	0.36(0.16,0.82)	0.02
**Cohabiting Women**				
Perceived Community Stigma (Y vs N)	1.56(0.88, 2.79)	0.13	—	—
Perceived Community Norms (Y vs N)	1.12(0.62,2.04)	0.71	—	—
Anticipated Stigma (Y vs N)	0.29(0.15,0.58)	0.0003	0.26(0.13,0.52)	0.0002
**Single (Unmarried Women)**				
Perceived Community Stigma (Y vs N)	0.53(0.29,0.97)	0.04	0.41(0.20,0.82)	0.01
Perceived Community Norms (Y vs N)	1.16(0.61,2.24)	0.65	—	—
Anticipated Stigma (Y vs N)	0.31(0.16,0.59)	0.0004	0.25(0.11,0.57)	0.0009
**Married Women**				
Perceived Community Stigma (Y vs N)	1.60(0.83,3.11)	0.16	—	—
Perceived Community Norms (Y vs N)	1.06(0.54,2.08)	0.86	—	—
Anticipated Stigma (Y vs N)	0.28(0.13,0.60)	0.001	0.22(0.10,0.50)	0.0003

These results were presented in abstract form at the 8^th^ Conference on HIV Pathogenesis, Treatment and Prevention (IAS 2015). [41]

## Discussion

For women living with HIV, we found that the decision to disclose one’s status was associated with perceived community-level influences as well as individual-level psychological, clinical and relationship factors. Among all women in this study, individual-level factors (*anticipated stigma* and severe symptoms of depression) were deterrents to status disclosure. Older women were also less likely to disclose their status. Among unmarried women and those not living with their primary sexual partner, *perceived community HIV stigma* was significantly associated with lower likelihood of status disclosure. Our findings suggest that in addition to addressing mental health needs and anticipated stigma, developing community-oriented stigma-reduction interventions may also be useful to increase status disclosure and improve prevention efforts among women living with HIV.

Prior research has identified *anticipated stigma* as an important predictor of non-disclosure of serostatus to sexual partners. [42–45] In this study, *anticipated stigma* was measured using items that assessed fear of enacted stigma or discrimination in the form of rejection and violence. Though the items did not specifically address fear of disclosure within intimate relationships, they assessed the potential of discrimination post-disclosure broadly (inclusive of sexual partners). Not surprisingly, a large number of women in this cohort (40.9%) endorsed *anticipated stigma*, as defined. Zambian women reported the lowest rate of *anticipated stigma* (23%), possibly due to the generalized nature of the HIV epidemic in that country. *Anticipated stigma* in the form of fear of violence may have been particularly relevant for the women studied given that many (42%) reported a past history of intimate partner violence (IPV). These findings may have significant personal consequences for women living with HIV as well as increase their risk for transmission to their HIV negative sexual partners. Research on *anticipated stigma* has demonstrated that increased fear of discrimination leads to psychological distress, decreased quality of life, and poor retention in care. [46–48]

Experiencing symptoms of depression was also found to independently predict non-disclosure of serostatus to sexual partners in this study. Depression is common among women living with HIV, and many women (66%) reported symptoms ranging from mild to severe. [49,50] Previous research has demonstrated a consistent association between depression and non-disclosure. [51,52] Individuals living with HIV who express symptoms of depression often suffer from internalized stigma which inhibits disclosure to sexual partners, as well as others. [53,54] This study also found that unmarried and non-cohabiting women were more likely to report symptoms of severe depression and less likely to disclose to their sexual partners. These findings may indicate that depressive symptoms may in part be due to lack of emotional or economic support systems. Irrespective of the cause, untreated depression may lead to poor retention in care and non-adherence to antiretroviral therapy which can have dire health consequences for women living with HIV. [55,56]

Age was also found to be associated with non-disclosure. Older women were less likely to disclose to their sexual partners than those in the youngest age group included in this study (ages 18–24). In a review of disclosure among pregnant and post-partum women, younger age was found to be a consistent predictor of disclosure. [57] Many interventions have been developed to address disclosure and male partner engagement during pregnancy to support prevention of mother to child transmission of HIV. Though most of the women in this study were not pregnant at baseline, it is possible that younger participants who are of childbearing age may have received disclosure support prior to this assessment. This finding speaks to the need for disclosure support across the age spectrum among sexually active women.

Though most research on stigma among people living with HIV assesses its determinants on an individual-level, stigma does not begin within the individual. Discriminatory norms and beliefs are constructed and perpetuated within communities, institutions and society-at-large. Stigmatizing beliefs are subsequently perceived and internalized by individuals. Although efforts have been undertaken in all three countries to decrease negative perceptions about people living with HIV, this study found that 45% of all women perceived that their communities hold stigmatizing beliefs about people living with HIV. [58,59] We hypothesized that *perceived community HIV stigma* and *perceived community gender norms* would independently predict disclosure to sexual partners among women living with HIV. This theory did not hold for the entire cohort of women studied. However, among women who were unmarried and not cohabiting with their primary sexual partner, *perceived community HIV stigma* was a significant predictor of non-disclosure. While all women living with HIV may be exposed to stigmatizing beliefs held by their communities, women who are married or are cohabiting with their partner may have additional emotional and social support that serve as buffers against community-level discrimination. Unmarried and non-cohabiting women may also be more concerned about community stigma and more fearful of disclosure because of the potential of loss of future sexual partners. Interestingly, *perceived community gender norms* did not predict disclosure for any group of women in this study. This finding may be due to development of the construct; two of the three items used to measure *perceived community gender norms* (“there is an obligation to have children and therefore engage in unprotected (condomless) sex” and “I will lose my status in my community if I am not a mother or a father”) relate to procreation, and few women (17%) reported the desire to have additional children.

Though the geographical setting of this cohort and the women studied were diverse, this study found no differences in the frequency of disclosure to sexual partners over time between countries. Few studies have been designed to compare disclosure in multiple countries. In a review of the published literature on disclosure among people living with HIV living in diverse geographical settings, disclosure to sexual partners among women varied widely from 17%-90%. [60] In this study, anticipated stigma was found to be a significant predictor of non-disclosure for all women and *perceived community HIV stigma* was a predictor of non-disclosure for unmarried and non-cohabiting women. Previous research has noted that HIV stigma persists across countries. [61] Therefore, the similarity in disclosure across the three countries in this analysis may not be so surprising. However, caution should be exercised in making cross country comparisons due to dissimilarities in the socio-demographic characteristics of the cohorts compared, varying recruitment strategies (described elsewhere), and culture norms regarding reporting of disclosure and engagement in research. [34]

This study has several limitations. Recruitment of participants was non-random, and recruitment procedures varied between sites. [34] HPTN 063 only included 299 heterosexual women, therefore, our sample size is limited. To increase our statistical power, we conducted a longitudinal analysis which measured outcomes at five time points (baseline, 3, 6, 9 and 12 months). In our dataset, we observed disproportional missing responses for outcome across three sites over time. To adjust for potential bias caused by this missing pattern, we applied the inverse probability weighting technique with the inverse proportion of observations within each site at each visit as the weight for each observation within the site at that visit. The inverse probability weighting method is unbiased and efficient under an assumption that data are missing at random. The limitation of this method is that if the missing at random assumption does not hold, i.e. the missing data are related to unobserved outcomes, then the method would produce biased result. [62] In addition, all of the measures included in this study were self-reported and subject to recall and social desirability biases. In regards to the assessment of community beliefs and norms, data were collected via close-ended questions. It is possible that additional perceived beliefs and norms that were not queried could impact disclosure to sexual partners. Also, we did not measure other types of individual level stigma, such as internalized stigma. As stated above, due to cultural differences and varying recruitment strategies, comparisons between countries must be made with caution.

Disclosure to sexual partners is an important HIV prevention strategy. Although we are more than 30 years into the HIV epidemic, pervasive stigma continues to promote discrimination and impede open and honest communication between sexual partners. This study found that individual-level stigma, community-level stigma, and depression inhibit disclosure among women living with HIV. Continued focus on de-stigmatizing HIV infection through community programming, such as media campaigns and health education, is necessary. [58,63] However, shifting widespread cultural beliefs and norms is challenging. Therefore, individual-level interventions to overcome stigma, address mental health needs and promote disclosure within intimate partnerships must also continue to be implemented.

## Supporting Information

S1 Table Loadings and communality estimates from principal component analysis using cultural questionnaire. (DOCX)

S2 Table Loadings and communality estimates from principal component analysis using anticipated stigma questionnaire. (DOCX)

S3 Table Baseline demographics. Available upon request.
(CSV)

S4 Table Longitudinal data. Available upon request.
(CSV)

S5 Table Data dictionary. Available upon request.
(DOCX)
