# Supplementary material for: Community Cultural Norms, Stigma and Disclosure to Sexual Partners among Women Living with HIV in Thailand, Brazil and Zambia (HPTN 063)
Source: PLoS One. 2016 May 6;11(5):e0153600. doi: 10.1371/journal.pone.0153600 (PMC4859553; doi:10.1371/journal.pone.0153600)
Supplement: S1 Table — (DOCX) [file pone.0153600.s001.docx]

**S1 Table. Loadings and communality estimates from principal component analysis using cultural questionnaire**

| **Items** | **Sexual mores** | **Gender Norms** | **Communality Estimates** |
| --- | --- | --- | --- |
| It is believed that people with HIV have contracted the disease because they have engaged in immoral behavior | 0.78 | 0.02 | 0.60 |
| People believe that women with HIV have engaged in prostitution | 0.85 | 0.13 | 0.74 |
| People believe that women with HIV have engaged in sex with many partners | 0.85 | -0.08 | 0.73 |
| Women are obligated to be submissive to their husbands, and therefore cannot ask their husbands to use condoms | 0.16 | 0.56 | 0.34 |
| There is an obligation to have children and therefore engage in unprotected sex | -0.06 | 0.68 | 0.47 |
| I will lose my status in my community if I am not a father or mother | -0.04 | 0.66 | 0.44 |

**Note: Total communality estimates =3.33**
