# Supplementary material for: Community Cultural Norms, Stigma and Disclosure to Sexual Partners among Women Living with HIV in Thailand, Brazil and Zambia (HPTN 063)
Source: PLoS One. 2016 May 6;11(5):e0153600. doi: 10.1371/journal.pone.0153600 (PMC4859553; doi:10.1371/journal.pone.0153600)
Supplement: S2 Table — (DOCX) [file pone.0153600.s002.docx]

**S2 Table. Loadings and communality estimates from principal component analysis using anticipated stigma questionnaire**

| **Items** | **Anticipated Stigma** | **Communality Estimates** |
| --- | --- | --- |
| I fear discrimination if I disclose my HIV status to others | 0.63 | 0.39 |
| I fear being kicked out of my house if I disclose my HIV positive status to others | 0.58 | 0.34 |
| I fear being kicked out of my community if I disclose my HIV positive status to others | 0.82 | 0.67 |
| I am afraid of violence if I disclose my HIV positive status to others | 0.81 | 0.65 |
| I am afraid of losing my job if I disclose my HIV status to my boss or others | 0.67 | 0.45 |

**Note: Total communality estimates =2.50**
