# Supplementary material for: Community Cultural Norms, Stigma and Disclosure to Sexual Partners among Women Living with HIV in Thailand, Brazil and Zambia (HPTN 063)
Source: PLoS One. 2016 May 6;11(5):e0153600. doi: 10.1371/journal.pone.0153600 (PMC4859553; doi:10.1371/journal.pone.0153600)
Supplement: S5 Table — Available upon request. (DOCX) [file pone.0153600.s005.docx]

| **Variable Names** | **Format** | **Description** |  | | |
| --- | --- | --- | --- | --- | --- |
| Site | 1=Thailand, 2=Brazil, 3=Zambia | Categorical variable | | | |
| Uid |  | Participant id | | | |
| age |  | Continuous variable | | | |
| agenew | 1='18-24'  2='25-44'  3='>=45'; | age category in years (categorical- derived) | | | |
|  |  |  |  | | |
| demmar | 1=never married, 2=married,  3=separated/divorced  4=widowed | What is your current marital status? |  | | |
|  |  |  |  | | |
| marital_status | 1= not married  2=married | categorical variable (derived);  if demmar=1,3 or 4 then marital_status =’Not married”  if demmar=2 then marital status=married | | | |
|  |  |  |  | | |
| demunem | 1=checked, 0=unchecked | unemployed |  | | |
| demmech | 1=checked, 0=unchecked | Electronics/mechanics |  | | |
| demacc | 1=checked, 0=unchecked | accounting |  | | |
| demstu | 1=checked, 0=unchecked | student |  | | |
| demteach | 1=checked, 0=unchecked | teacher |  | | |
| demfarm | 1=checked, 0=unchecked | Farming/agriculture |  | | |
| demart | 1=checked, 0=unchecked | artist |  | | |
| demret | 1=checked, 0=unchecked | retired |  | | |
| demdw | 1=checked, 0=unchecked | Domestic worker (cook, clean, child care) |  | | |
| demhw | 1=checked, 0=unchecked | Housewife(not for pay) |  | | |
| demrelw | 1=checked, 0=unchecked | Religious work |  | | |
| demmp | 1=checked, 0=unchecked | Military/police |  | | |
| demgv | 1=checked, 0=unchecked | government |  | | |
| demsw | 1=checked, 0=unchecked | Sex worker |  | | |
| demdoc | 1=checked, 0=unchecked | Health care (eg. Doctor, nurse) |  | | |
| demoffc | 1=checked, 0=unchecked | Business(eg. Manager, office work) |  | | |
| demskl | 1=checked, 0=unchecked | Skilled labor (eg. Required training) |  | | |
| demdrv | 1=checked, 0=unchecked | driver |  | | |
| demuskl | 1=checked, 0=unchecked | Unskilled labor (no training) |  | | |
| demwoth | 1=checked, 0=unchecked | other |  | | |
| demwothx |  | Other listing |  | | |
| Employment | 1=unemployed  2=employed outside home  3=sex worker | (categorical variable- derived)  Employment status  if demunem=1  Employment=1;  if demstu=1  Employment=1;  if demhw=1  Employment=1;  if demmech=1  Employment=2;  if demacc=1  Employment=2;  if demteach=1  Employment=2;  if demfarm=1  Employment=2;  if demart=1  Employment=2;  if demret=1  Employment=2;  if demdw=1  Employment=2;  if demrelw=1  Employment=2;  if demmp=1  Employment=2;  if demgv=1  Employment=2;  if demdoc=1  Employment=2;  if demoffc=1  Employment=2;  if demskl=1  Employment=2;  if demdrv=1  Employment=2;  if demuskl=1  Employment=2;  if demwoth=1  Employment=2;  if demsw=1  Employment=3; |  | | |
| demedu | 1=no schooling, 2=primary school, not complete, 3=primary school, complete,  4=secondary, not complete,  5=secondary, complete  6=technical training, not complete  7=technical training, complete  8=college or university, not complete  9=college or university, complete | What is the participant’s highest level of education? |  | | |
|  |  |  |  | | |
| demedunew | 1='No schooling'  2='Primary school'  3='More than primary school' | Highest level of education(derived);  If demedu=1 then demedunew=no schooling  If demedu=2 or 3 then demedunew=primary school  If demedu=4,5,6,7,8,or 9 then demedunew=more than primary school | | | |
|  |  |  |  | | |
| demmkids | 1='Yes'  2=no  3=Don’t know | Desire for Biological Children(categorical) | | | |
|  |  |  |  | | |
| enrphit | 1=yes  2=no | Have you ever had a sexual partner (including a current or former spouse,boy/girlfriend or other sexual partner) who has hit,slapped,kicked,pushed,shoved or otherwise physically hurt you? |  | | |
|  |  |  |  | | |
| enrprape | 1=yes  2=no | Has anyone (including current or former spouse,boy/girlfriend or other sexual partner) ever tried to force you to have sex when you didn’t want to? |  | | |
|  |  |  |  | | |
| past | 1='yes' 2='no'; | past history of physical abuse or sexual violence (categorical)  if enrphit=1 or enrprape=1 then past=1;  if enrphit=2 and enrprape=2 then past=2; | | | |
|  |  |  |  | | |
| Fu3phit | 1=yes  2=no | In the past 3 months,did a sexual partner (including a current or former spouse, boy/girlfriend, or other sexual partner) hit, slap,kick, push, shove or otherwise physically hurt you? |  | | |
|  |  |  |  | | |
| Fu3rape | 1=yes  2=no | In the past 3 months, did anyone (including a current or former spouse, boy/girlfriend, or other sexual partner) try to force you to have sex when you didn’t want to? |  | | |
|  |  |  |  | | |
| recent | 1='yes'  2=no | recent history of physical abuse or sexual violence (categorical)  if fu3phit=1 or fu3rape=1 then recent=1;  if fu3phit=2 and fu3rape=2 then recent=2; | | | |
|  |  |  |  | | |
| Alc_score |  | Score given to alcohol consumption (continuous variable) |  | | |
|  |  |  |  | | |
| etoh | 1=yes  2=no | Categorical variable(derived)  Alcohol abuse  If alc_score greater than 8 then etoh=1;  If alc_score less than or equal to 8 then etoh=2; |  | | |
|  |  |  |  | | |
| saudrugs | 1=yes  2=no | Have you used any drugs in the past 3 months? This includes anything you may have smoked, injected, inhaled, swallowed, huffed or snorted |  | | |
| abuse | 1='yes'  2=no | Drugs or alcohol abuse in the past three months (categorical-- derived)  If etoh=1 or saudrugs=1 then abuse=1;  Else abuse=2; | | | |
|  |  |  |  | | |
| cesd | 1 = " Normal "  2 = " Mild to Moderate Depression "  3 = " Major depression "; | Depression(categorical) | | | |
|  |  |  |  | | |
| spqrpart | 1='yes'  2=no | Currently sexually active (categorical) | | | |
|  |  |  |  | | |
| spqmorep | 1=none  2=one  3=two  4=three  5=four or more | How many regular sexual partners do you currently have other than the primary partner we already discussed? |  | | |
|  |  |  |  | | |
| Partners_num |  | Continuous variable (derived)  Number of regular sexual partners  if spqrpart=1 and spqmorep=1 then partners_num=1  if spqmorep=2 then partners_num=1  if spqmorep=3 then partners_num=2;  if spqmorep=4 then partners_num=3;  if spqmorep=5 then partners_num=4; |  | | |
|  |  |  |  | |  |
| spqliv | 1='yes' | Cohabiting with primary sexual partners (categorical) | | |  |
|  | 2='no'; |  | |  |  |
|  |  |  | |  |  |
| spqpstat | 1=HIV negative  2=confirmed HIV positive  3=don’t know | What is the current HIV status of your current partner? | |  |  |
|  |  |  | |  |  |
| Spqptest | 1=within the last three months  2=more than 3 months to 6 months ago  3=more than 6 months to a year ago  4=more than 1 year to 3 years ago  5=more than 3 years ago  6=don’t know  7=never been tested | When was the last time your primary partner was tested for HIV? | |  |  |
|  |  |  | |  |  |
| Partner_status | 1=HIV positive  0=HIV negative or unknown | Categorical variable (derived)  If spqpstat=1 or 3 or spqtest=6 or 7 then partner_status=0;  If spqpstat=2 then partner_status=1; | |  |  |
|  |  |  | |  |  |
| AAarv | 1=yes  2=no | Have you ever taken ARV for your HIV? This doesn’t include vitamins, herbs or other medications that you may take to feel better | |  |  |
|  |  |  | |  |  |
| AAtake | 1=very poor  2=poor  3=fair  4=good  5=very good  6=excellent | Thinking about the last 3 months, on average ,how would you rate your ability to take all your ARV as your doctor prescribed | |  |  |
|  |  |  | |  |  |
| ability_arv | 1=poor or very poor  2=fair  3=good, very good or excellent | (categorical variable---derived)  Ability to take all ARV (last 3 months)  If aatake=1 or 2 then ability_arv=1;  If aatake=3 then ability_arv=2;  If aatake=4,5 or 6 then ability_arv=3 | |  |  |
|  |  |  | |  |  |
| cdvcd |  | (Continuous variable)  Absolute CD4+ count | |  |  |
|  |  |  | |  |  |
| enrdt | Date | Enrollment date | |  |  |
| enrdxdt | Date | When you were first diagnosed with HIV? | |  |  |
|  |  |  | |  |  |
| diagnosis |  | Continuous variable (derived)  Time since HIV diagnosis (days)  Enrdt-enrdxdt | |  |  |
|  |  |  | |  |  |
| enrpreg | 1=yes  2=no | Are you currently pregnant? | |  |  |
|  |  |  | |  |  |
| visit | 0='Baseline'  3='Three months'  6='Six months'  9='Nine months'  12='Twelve months'; | Quarterly visits | |  |  |
|  |  |  | |  |  |
| ipw |  | Continuous variable  Weights calculated using inverse probability weighting method | |  |  |

**Description of variables used in Principal Component Analysis (PCA) from cultural questionnaire**

| **Variables** | **Format** | **Description** |
| --- | --- | --- |
| cqhivim | 1=strongly disagree  2=disagree  3=agree  4=strongly agree | It is believed that people with HIV have contracted the disease because they have engaged in immoral behavior |
| cqwpros | 1=strongly disagree  2=disagree  3=agree  4=strongly agree | People believe that women with HIV have engaged in prostitution |
| cqwprom | 1=strongly disagree  2=disagree  3=agree  4=strongly agree | People believe that women with HIV have engaged in sex with many partners |
| Cqwsub | 1=strongly disagree  2=disagree  3=agree  4=strongly agree | Women are obligated to be submissive to their husbands, and therefore cannot ask their husbands to use condoms |
| cqkids | 1=strongly disagree  2=disagree  3=agree  4=strongly agree | There is an obligation to have children, and therefore engage in unprotected sex |
| cqlsstat | 1=strongly disagree  2=disagree  3=agree  4=strongly agree | I will lose my status in my community if I am not a father or mother |
| Factor1 |  | First factor obtained using PCA (continuous variable) |
| Factor2 |  | Second factor obtained using PCA (continuous variable) |
| Sexual_mores | 1=positive scores  0=negative scores | Categorical variable (derived)  Sexual mores/behavior  If factor1 greater than 0 then sexual_mores=positive scores  If factor1 less than 0 then sexual_mores=negative scores |
| Marital_norms | 1=positive scores  0=negative scores | Categorical variable (derived)  Marital norms/procreation  If factor2 greater than 0 then marital_norms=positive scores  If factor2 less than 0 then marital_norms=negative scores |

**Description of variables used in Principal Component Analysis (PCA) for anticipated stigma**

| **Variables** | **Format** | **Description** |
| --- | --- | --- |
| cqfdisc | 1=strongly disagree  2=disagree  3=agree  4=strongly agree | I fear discrimination if I disclose my HIV status to others |
| cqfhless | 1=strongly disagree  2=disagree  3=agree  4=strongly agree | I fear being kicked out of my house if I disclose my HIV positive status to others |
| cqfcless | 1=strongly disagree  2=disagree  3=agree  4=strongly agree | I fear being kicked out of my community if I disclose my HIV positive status to others |
| Cqfviol | 1=strongly disagree  2=disagree  3=agree  4=strongly agree | I am afraid of violence if I disclose my HIV positive status to others |
| cqfjob | 1=strongly disagree  2=disagree  3=agree  4=strongly agree | I am afraid of losing my job if I disclose my HIV status to my boss or others |
| Factor3 |  | Factor obtained using PCA (continuous variable) |
| stigma | 1=positive scores  0=negative scores | Categorical variable (derived)  Perceived HIV stigma  If factor3 greater than 0 then stigma=positive scores  If factor3 less than 0 then stigma=negative scores |

| **Outcome variable** | **Format** | **Description** |
| --- | --- | --- |
| actllpt | 1=I have not had anal or vaginal sex in the past 3 months  2=They already knew I was HIV-positive  3=I did not tell any of them  4=I told less than half of them  5=I told about half of them  6=I told more than half of them  7=I told all of them | Thinking about the people that you have had anal or vaginal sex within the past 3 months, how often have you told them that you are HIV-positive? |
|  |  |  |
| acdisclose | 1=disclosed to some or all  0=disclosed to none | Disclosure status (derived)  If actllpt=2,4,5,6,or 7 then acdisclose=1  If actllpt=1 or 3 then acdisclose=0 |
